# Supplementary material for: Results and Clinical Interpretation of Germline RET Analysis in a Series of Patients with Medullary Thyroid Carcinoma: The Challenge of the Variants of Uncertain Significance
Source: Cancers (Basel). 2020 Nov 5;12(11):3268. doi: 10.3390/cancers12113268 (PMC7694403; doi:10.3390/cancers12113268)
Supplement: Supplementary file 1 [file cancers-12-03268-s001.pdf]

cancers

# Supplementary Materials: Results and Clinical Interpretation of Germline RET Analysis in A Series of Patients with Medullary Thyroid Carcinoma: The Challenge of the Variants of Uncertain Significance.

Giovanni Innella, Cesare Rossi, Maria Romagnoli, Andrea Repaci, Davide Bianchi and Maria Elena Cantarini, Davide Martorana, Lea Godino, Andrea Pession, Antonio Percesepe, Uberto Pagotto and Daniela Turchetti*

**Table S1.** Case series under study.

| Patient number | Family ID | Family phenotype | Affected/Healthy | Type of analysis | *RET* variant |
|---|---|---|---|---|---|
| 1 | 10-O-02 | MTC | A | C | NO |
| 2 | 28-O-03 | MTC | A | C | NO |
| 3 | 91-O-03 | MTC | A | C | YES |
| 4 | 91-O-03 | MTC | A | T | YES |
| 5 | 31-O-04 | MTC | A | C | NO |
| 6 | 144-O-04 | MTC | A | C | YES |
| 7 | 59-M-04 | MTC | A | C | NO |
| 8 | 91-O-03 | MTC | A | T | YES |
| 9 | 91-O-03 | MTC | A | T | YES |
| 10 | 91-O-03 | MTC | H | T | YES |
| 11 | 91-O-03 | MTC | H | T | NO |
| 12 | 91-O-03 | MTC | H | T | NO |
| 13 | 27-O-05 | MTC | A | C | NO |
| 14 | 91-O-03 | MTC | H | T | YES |
| 15 | 91-O-03 | MTC | A | T | YES |
| 16 | 91-O-03 | MTC | H | T | NO |
| 17 | 42-O-07 | MTC | A | C | NO |
| 18 | 9-O-08 | MTC | H | T | YES |
| 19 | LAB-01 | UK | UK | C | NO |
| 20 | LAB-02 | UK | UK | C | NO |
| 21 | LAB-03 | IG | UK | C | NO |
| 22 | 9-O-08 | MTC | H | T | NO |
| 23 | 9-O-08 | MTC | H | T | YES |
| 24 | 91-O-03 | MTC | H | T | NO |
| 25 | 19-O-09 | MTC | A | C | NO |
| 26 | LAB-04 | UK | A | C | NO |
| 27 | 21-O-09 | MTC | A | C | NO |
| 28 | 31-O-09 | MTC | A | C | NO |
| 29 | 54-O-09 | MTC | A | C | YES |
| 30 | 54-O-09 | MTC | A | T | YES |
| 31 | 42-O-10 | MTC | A | C | NO |

| | | | | | |
|---|---|---|---|---|---|
| 32 | 91-O-03 | MTC | H | T | NO |
| 33 | 13-O-10 | MTC | A | C | NO |
| 34 | 44-O-10 | MTC | A | C | NO |
| 35 | 53-O-10 | MTC | A | C | NO |
| 36 | 63-O-10 | MTC | A | C | NO |
| 37 | 54-O-09 | MTC | H | T | YES |
| 38 | 54-O-09 | MTC | H | T | YES |
| 39 | 12-O-11 | MTC | A | C | NO |
| 40 | 13-O-11 | MEN2A | A | C | YES |
| 41 | 29-O-11 | MTC | A | C | NO |
| 42 | 33-O-11 | MTC | A | C | NO |
| 43 | 53-O-11 | MTC | A | C | NO |
| 44 | 67-O-11 | MTC | A | C | NO |
| 45 | 68-O-11 | MTC | A | C | YES |
| 46 | 92-O-11 | MTC | A | C | NO |
| 47 | 74-O-11 | CCH | A | C | NO |
| 48 | 87-O-11 | MTC | A | C | NO |
| 49 | 13-O-12 | MTC | A | C | NO |
| 50 | 54-O-11 | MTC | A | C | NO |
| 51 | 35-O-12 | CCH | A | C | NO |
| 52 | 190-O-13 | PHPT | A | C | NO |
| 53 | 190-O-13 | PHPT | A | C | NO |
| 54 | 58-O-12 | MTC | A | C | NO |
| 55 | 65-O-12 | MTC | A | C | NO |
| 56 | 100-O-12 | MTC | A | C | NO |
| 57 | LAB-05 | PHPT | UK | C | NO |
| 58 | 228-O-18 | MTC | A | C | YES |
| 59 | LAB-06 | PGL/PCC | A | C | NO |
| 60 | 58-O-13 | MTC | A | C | NO |
| 61 | 53-O-13 | PHPT | A | C | NO |
| 62 | 72-O-13 | MTC | H | T | YES |
| 63 | 136-O-13 | PHPT | A | C | YES |
| 64 | 81-O-13 | MTC | A | C | NO |
| 65 | 89-O-13 | MTC | A | C | NO |
| 66 | 54-O-09 | MTC | H | T | YES |
| 67 | 103-O-13 | MTC | A | C | NO |
| 68 | LAB-07 | PHPT | UK | C | NO |
| 69 | 121-O-13 | MTC | A | C | NO |
| 70 | 228-O-18 | MTC | A | T | YES |
| 71 | 228-O-18 | MTC | A | T | YES |
| 72 | 228-O-18 | MTC | H | T | YES |
| 73 | 228-O-18 | MTC | A | T | YES |
| 74 | 228-O-18 | MTC | H | T | NO |

| | | | | | |
|---|---|---|---|---|---|
| 75 | 10-O-14 | MTC | A | C | NO |
| 76 | 81-O-14 | MTC | A | C | NO |
| 77 | 228-O-18 | MTC | H | T | YES |
| 78 | 228-O-18 | MTC | H | T | NO |
| 79 | 228-O-18 | MTC | H | T | NO |
| 80 | 144-O-14 | MTC | A | C | NO |
| 81 | 168-O-14 | MTC | A | C | YES |
| 82 | 175-O-14 | MTC | A | C | NO |
| 83 | LAB-08 | PTC | UK | C | NO |
| 84 | 204-O-14 | MTC | A | C | NO |
| 85 | 168-O-14 | MTC | H | T | YES |
| 86 | 120-O-14 | PGL/PCC | A | C | NO |
| 87 | LAB-09 | UK | UK | C | NO |
| 88 | LAB-10 | UK | UK | C | NO |
| 89 | 269-O-14 | MTC | A | C | NO |
| 90 | LAB-11 | CCH | UK | C | NO |
| 91 | 168-O-14 | MTC | H | T | YES |
| 92 | 168-O-14 | MTC | H | T | YES |
| 93 | 168-O-14 | MTC | H | T | NO |
| 94 | LAB-12 | UK | UK | T | YES |
| 95 | LAB-13 | PHPT | UK | C | NO |
| 96 | 31-O-15 | PGL/PCC | A | C | NO |
| 97 | 39-O-15 | MTC | A | C | NO |
| 98 | 45-O-15 | PGL/PCC | A | C | NO |
| 99 | 53-O-15 | PGL/PCC | A | C | NO |
| 100 | 89-O-15 | MTC | A | C | YES |
| 101 | 89-O-15 | MTC | H | T | NO |
| 102 | 89-O-15 | MTC | H | T | YES |
| 103 | 89-O-15 | MTC | H | T | YES |
| 104 | 89-O-15 | MTC | H | T | YES |
| 105 | 169-O-15 | MEN2B | A | C | YES |
| 106 | 169-O-15 | MEN2B | H | T | NO |
| 107 | 169-O-15 | MEN2B | H | T | NO |
| 108 | 169-O-15 | MEN2B | H | T | NO |
| 109 | 169-O-15 | MEN2B | H | T | NO |
| 110 | 169-O-15 | MEN2B | H | T | NO |
| 111 | 32-O-15 | MTC | A | C | NO |
| 112 | 70-O-15 | PGL/PCC | A | C | NO |
| 113 | 219-O-15 | PGL/PCC | A | C | NO |
| 114 | 316-O-15 | PGL/PCC | A | C | NO |
| 115 | 13-O-16 | MTC | A | C | NO |
| 116 | 121-O-16 | PGL/PCC | A | C | NO |
| 117 | LAB-14 | UK | UK | C | NO |

| | | | | | |
|---|---|---|---|---|---|
| 118 | LAB-15 | UK | UK | C | NO |
| 119 | LAB-16 | UK | UK | C | YES |
| 120 | LAB-17 | UK | UK | C | NO |
| 121 | 204-O-16 | PGL/PCC | A | C | NO |
| 122 | 275-O-16 | MTC | A | C | NO |
| 123 | 371-O-16 | CCH | A | C | NO |
| 124 | 166-O-17 | MTC | A | C | NO |
| 125 | 191-O-17 | MTC | H | T | YES |
| 126 | 288-O-17 | PGL/PCC | A | C | NO |
| 127 | 359-O-17 | PGL/PCC | A | C | NO |
| 128 | 1-MAG-CRC | PGL/PCC | A | C | NO |
| 129 | LAB-18 | UK | UK | C | NO |
| 130 | 43-O-18 | MTC | A | C | NO |
| 131 | 55-O-18 | MTC | A | C | YES |
| 132 | 55-O-18 | MTC | H | T | NO |
| 133 | LAB-19 | UK | UK | C | NO |
| 134 | LAB-20 | MEN2B | A | C | YES |
| 135 | LAB-20 | MEN2B | H | T | NO |
| 136 | LAB-20 | MEN2B | H | T | NO |
| 137 | 55-O-18 | MTC | H | T | NO |
| 138 | 55-O-18 | MTC | H | T | NO |
| 139 | LAB-21 | UK | UK | C | YES |
| 140 | LAB-21 | UK | UK | T | NO |
| 141 | LAB-21 | UK | UK | T | NO |
| 142 | 142-O-18 | MEN2A | A | T | YES |
| 143 | 142-O-18 | MEN2A | H | T | NO |
| 144 | 142-O-18 | MEN2A | H | T | NO |
| 145 | LAB-22 | UK | UK | C | NO |
| 146 | 228-O-18 | MTC | A | T | YES |
| 147 | 228-O-18 | MTC | H | T | YES |
| 148 | 228-O-18 | MTC | H | T | NO |
| 149 | 228-O-18 | MTC | H | T | NO |
| 150 | 228-O-18 | MTC | A | T | YES |
| 151 | LAB-23 | UK | UK | C | NO |
| 152 | LAB-24 | UK | UK | C | NO |
| 153 | LAB-25 | UK | UK | C | NO |
| 154 | LAB-26 | UK | UK | C | NO |
| 155 | LAB-27 | UK | UK | T | NO |
| 156 | LAB-28 | UK | UK | C | NO |
| 157 | 228-O-18 | MTC | H | T | NO |
| 158 | 520-M-18 | MTC | A | C | NO |
| 159 | 324-O-19 | MTC | A | C | NO |
| 160 | 330-O-19 | PGL/PPC | A | C | NO |

| | | | | | |
|---|---|---|---|---|---|
| 161 | 83-OB-19 | PHPT | A | C | NO |
| 162 | 115-O-20 | MTC | H | T | YES |
| 163 | 91-O-03 | MTC | H | T | YES |

Abbreviations: MTC = Medullary Thyroid Carcinoma; UK = Unknown; IG = Intestinal Ganglioneuromatosis; MEN2A = Multiple Endocrine Neoplasia type 2 A; CCH = C Cell Hyperplasia; PGL/PCC = Paraganglioma/Pheocromocytoma; PHPT = Primary Hyperparathyroidism; MEN2B = Multiple Endocrine Neoplasia type 2 B; C = Complete analysis; T = Targeted analysis
